# Three-dimensional ultrasound in fetal medicine after 25 years in clinical practice: many advances and some questions

**DOI:** 10.1590/0100-3984.2016.49.5e1

**Published:** 2016

**Authors:** Edward Araujo Júnior

**Affiliations:** 1 Tenured Adjunct Professor of Fetal Medicine in the Department of Obstetrics of the Escola Paulista de Medicina da Universidade Federal de São Paulo (EPM-Unifesp), São Paulo, SP, Brazil. E-mail: araujojred@terra.com.br.

The first three-dimensional ultrasound system (3D-US) in obstetrics was proposed by Baba et al.^(1)^ in 1986 and consisted of a mechanical arm attached to a two-dimensional convex probe that was able to capture two-dimensional images, which were sent to a computer and processed into three-dimensional volumes. However, the long time required for processing and the low quality of the images impeded their clinical application.

In the early 1990s, the advent of automatic scanning volumetric probes opened new horizons in the three-dimensional evaluation of the fetus. In 1992, Kuo et al.^(2)^ demonstrated the first application of 3D-US fetal evaluation. Since then, various applications of 3D-US in fetal medicine have been described, such as the evaluation of fetal malformations^(3)^, volumetric assessment of fetal organs^(4)^, cardiac assessment^(5)^, evaluation of the central nervous system^(6)^, and the use of power Doppler^(7)^. More recently, new software, such as HDlive, allow a realistic vision of the face and surfaces of the fetus^(8)^. The main advantages of 3D-US would be the following: evaluation of a fetal structure simultaneously in the three orthogonal planes; fetal assessment in the absence of the mother; less reliance on operator skills; and the possibility of sending three-dimensional volumes for analysis at tertiary care centers^(9)^.

At 25 years after the introduction of 3D-US in the clinical practice of fetal medicine, despite the greater availability of devices that are faster and produce images with higher resolution, as well as the involvement of examiners who are more experienced in the technique, questions remain about the real advantage in relation to the two-dimensional ultrasound (2D-US) for the maternal-fetal dyad.

In this issue of **Radiologia Brasileira**, Werner et al.^(10)^ present the experience of their group in the construction of virtual and physical 3D models of 26 singleton fetuses and 5 twin fetuses with various malformations, obtained through 3D-US, magnetic resonance imaging (MRI), and computed tomography (CT). The authors state that additive manufacturing technology allows the conversion of a virtual 3D model to a physical model, with precise dimensions, in a process that is fast and easy. They conclude that the physical 3D models allow greater interaction between the parent(s) and the fetus, as well as representing a method of continuing medical education. Since 2010, this research group has conducted a number of studies proposing the use of virtual and physical 3D models-based on data obtained with 3D-US, MRI, and CT-in the evaluation of various fetal malformations^(11,14)^, intrauterine infections ^(15,16)^ and maternal-fetal interaction in blind couples^(17)^. In all of those studies, the 3D models have allowed a better understanding of fetal disease by the medical team and by the parents, as well as enabling greater maternal-fetal interaction. In addition, the virtual 3D models enabled the virtual navigation by the fetal trachea in cases of cervical teratoma, in order to assess the degree of compression in the prenatal period and enable better management in childbirth and postpartum^(18)^. Although the printing costs of the physical 3D models make their use currently accessible only to a small portion of the population, we believe that more rapid technological development and broader dissemination of the method will soon make this diagnostic tool available to a larger portion of the population.

In summary, after 25 years of clinical practice of 3D-US in fetal medicine, various advances were made in prenatal diagnosis. However, certain questions remain unanswered. The answers to those questions could arise along with ongoing technological development. The virtual and physical 3D models allow a new form of fetal assessment by medical staff, in addition to increasing maternal-fetal interaction, especially in cases of blind couples.
